# Supplementary material for: Misspelled‐Word Reading Modulates Late Cortical Dynamics
Source: Hum Brain Mapp. 2025 Jun 12;46(8):e70247. doi: 10.1002/hbm.70247 (PMC12159769; doi:10.1002/hbm.70247)
Supplement: Supplementary file 1 — Figure S1. Group‐level source estimates (MNE‐dSPM) for each condition in four selected time windows. Figure S2. a, ROIs in the right hemisphere, and averaged evoked responses of each category within the ROIs. Solid bars under the plots indicate the time clusters associated with p < 0.05 based on cluster‐based permutation tests between misspelled words and real words. b, One‐way repeated measures ANOVA and pairwise t‐test (FDR corrected) between conditions in each time window. Asterisks above each heatmap indicate the significance level obtained from ANOVA (***p < 0.001; **p < 0.01; *p < 0.05). [file HBM-46-e70247-s001.pdf]

Supplementary materials

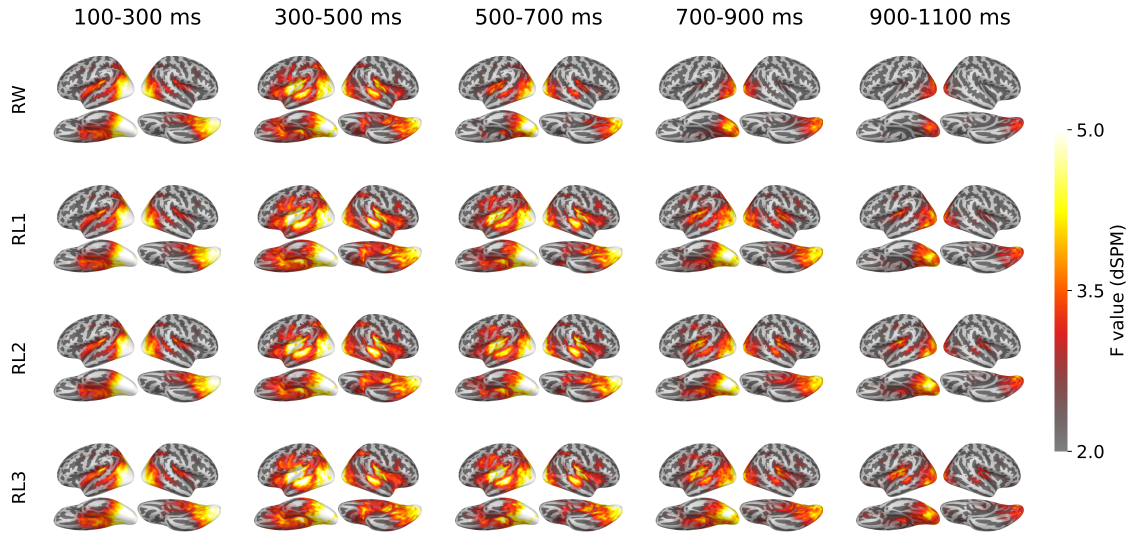

Supplementary Figure 1: Group-level source estimates (MNE-dSPM) for each condition in four selected time windows.

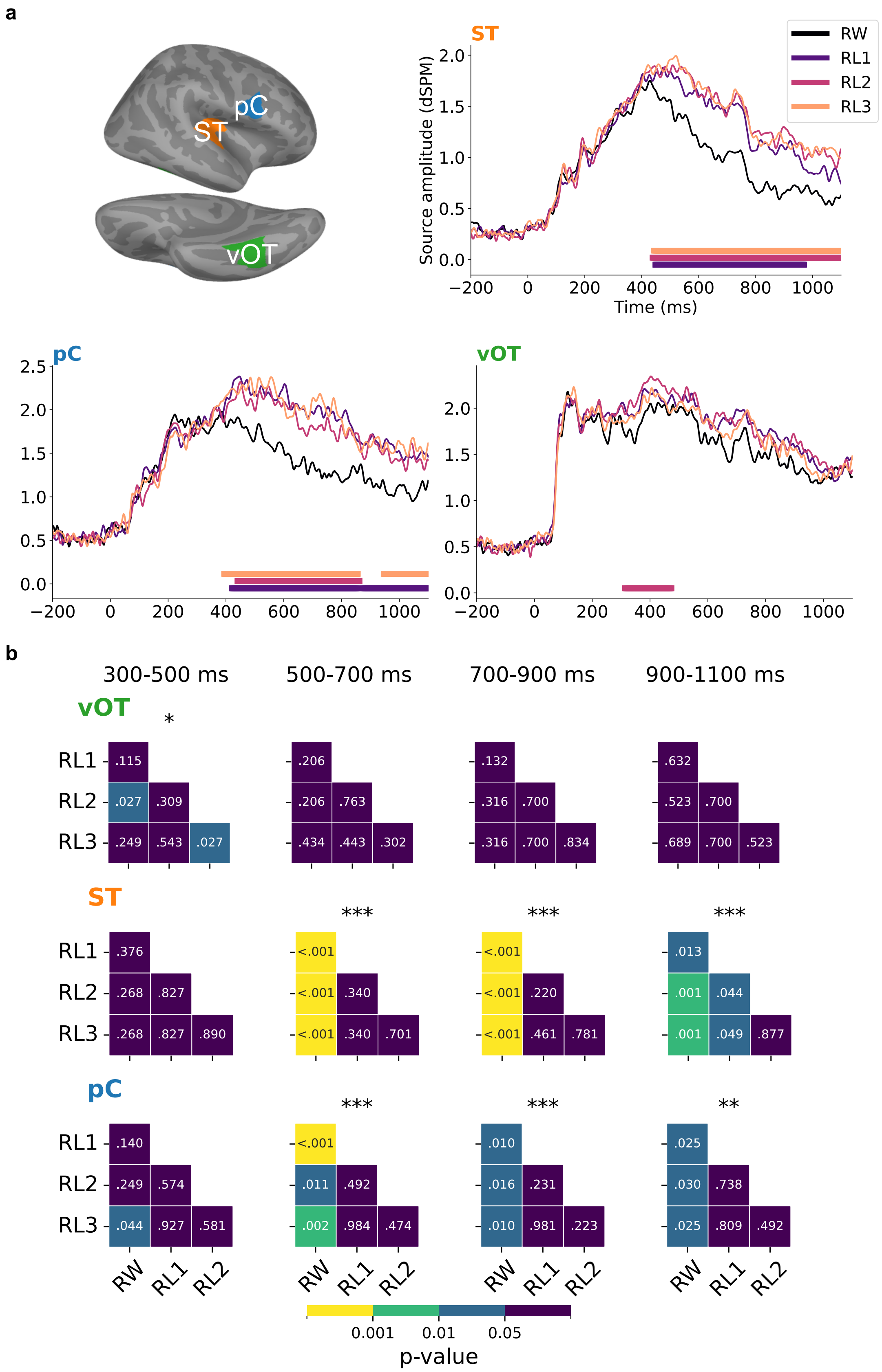

Supplementary Figure 2: **a**, ROIs in the right hemisphere, and averaged evoked responses of each category within the ROIs. Solid bars under the plots indicate the time clusters associated with $p < 0.05$ based on cluster-based permutation tests between misspelled words and real words. **b**, One-way repeated measures ANOVA and Pairwise t-test (FDR corrected) between conditions in each time window. Asterisks above each heatmap indicate the significance level obtained from ANOVA (***, $p < 0.001$; **, $p < 0.01$; *, $p < 0.05$).
